# OTME-3. Dissection of the role of stromal microenvironment and tumor-TME crosstalk in pediatric brain cancer

**DOI:** 10.1093/noajnl/vdab070.054

**Published:** 2021-07-05

**Authors:** Aniello Federico, Marcel Kool

**Affiliations:** 1 Hopp-Children’s Cancer Center (KiTZ), Heidelberg, Germany; 2 Division of Pediatric Neurooncology, German Cancer Research Center (DKFZ) and German Cancer Consortium (DKTK), Heidelberg, Germany

## Abstract

Brain tumors are the deadliest malignancies that occur during childhood and strong efforts are required to develop innovative therapeutic strategies. The intrinsic capacity of malignant cells to organize, shape and exploit the surrounding environment where they develop (tumor microenvironment, TME), has not been fully elucidated for pediatric brain cancers yet. Here, we exploited a multi-omic approach to define the TME cell populations and their contributions in the most common pediatric brain tumor entities, such as medulloblastomas and ependymomas. Analysis of single-cell RNA sequencing data of human tumors resulted in the identification of heterogeneous populations of non-malignant cells present in the TME. In particular, re-clustering and marker-based cell type assignment strategies allowed to define a broad range of immune and stromal subclasses showing distinctive expression signatures reflecting variegated functional roles. By cross-matching the tumor data with normal brain expression atlases, we could further refine the annotation of the newly identified stromal functional subpopulations and define the “tumor-associated” marker signatures of genes exclusively enriched in stromal cells within the TME, linked to immune activation, cell adhesion and cytokine regulation pathways. Bulk transcriptomic data of human tumors and matching patient-derived xenografts (PDXs) showed that a group of secreted stromal factors acting as regulators of tumorigenic mechanisms, such as IGF2 and COL4A1, are lost after xenografting and replaced by the host murine microenvironment, suggesting that tumor cells are involved in paracrine and bivalent crosstalk with TME cells, impacting on tumor cell growth and progression. Finally, bulk deconvolution and cell-cell communication analysis were exploited to define, respectively, the stromal cell proportions and the key factors involved in the tumor-TME crosstalk; this latter can be considered as possible targets for tailored and more specific anti-tumor therapeutic strategies.

